# Efficiency of a virtual fracture clinic review protocol in adult patients with distal radial fractures requiring semi-acute surgical treatment

**DOI:** 10.1007/s00068-025-02764-3

**Published:** 2025-02-07

**Authors:** Dorien A. Salentijn, Gijs J. A. Willinge, Ruben N. van Veen, Marcel G. W. Dijkgraaf

**Affiliations:** 1 Department of Trauma Surgery, OLVG, Jan Tooropstraat 164, Amsterdam, 1061 AE Netherlands; 2 Department of Epidemiology and Data Science, Amsterdam UMC - University of Amsterdam, Meibergdreef 9, Amsterdam, 1105 AZ Netherlands; 3 Amsterdam Public Health, Meibergdreef 9, Amsterdam, 1105 AZ Netherlands

**Keywords:** Distal radius fracture, Semi-acute surgery, VFC, ORIF, Efficiently

## Abstract

**Purpose:**

The aim of this study was to evaluate the effect of implementation of a Virtual Fracture Clinic (VFC) review protocol on the time between injury and surgery, and on secondary healthcare utilization, in patients with Distal Radius Fractures (DRFs) requiring semi-acute surgery.

**Methods:**

Data for this retrospective before-after study were gathered between April 2017 and March 2019 (Pre-VFC *n* = 269), and between April 2021 and March 2023 (VFC *n* = 440) in a large level 2 urban trauma center. The primary outcome was the number of days between injury and operation. Furthermore secondary healthcare utilization was assessed.

**Results:**

The average time between injury and surgery was 11.0 days (95% CI: 10.6–11.5) before and 9.2 days (95% CI: 8.9–9.6) after VFC-implementation ( *p* < 0.001). Following VFC-implementation, 33% (was 17%) of patients underwent surgery within 7 days, 92% (was 84%) within 2 weeks, and 99% (was 96%) within 3 weeks (*p* < 0.001). This included patients with delays of up to 15 days between injury and their initial hospital presentation. Hospital contacts decreased from 5 (IQR: 4–6) to 4 (IQR: 3–5) whereof physical consults decreased from 4 (IQR: 3–5) to 1 and telephone contacts increased from negligible to 1 (IQR: 1–2). Radiographs reduced from 6 (IQR: 5–7) to 4 (IQR: 3–5).

**Conclusions:**

Implementation of a VFC-review protocol is associated with a reduced time between injury and semi-acute surgery for DRFs and reflects an improvement in quality of timely planning. Secondary healthcare utilization is reduced and a shift to remote delivery of care is observed.

**Level of evidence:**

Level III.

**Supplementary Information:**

The online version contains supplementary material available at 10.1007/s00068-025-02764-3.

## Introduction

Distal radius fractures (DRFs) are the most prevalent fractures among individuals under the age of 75, with an incidence of approximately 32 per 10,000 persons per year [1, 2]. There is a growing tendency towards operative treatment using open reduction internal fixation (ORIF) aiming to achieve stable fracture fixation to facilitate early mobility [3–8]. There is no established benchmark for the optimal time between injury and surgery, but the preference is generally to operate sooner rather than later, ideally within a maximum of three weeks for this type of injury [9–13]. Due to the rising incidence and the growing tendency towards operative treatment, patients with DRFs are causing an increasing burden on the trauma care system. This potentially complicates timely scheduling of surgery and subsequent outpatient clinic appointments. This is further challenged by an overall increase of other traumatic injuries [14].

A solution to this problem could be found in more adequate and efficient planning through the introduction of a Virtual Fracture Clinic (VFC) review protocol [15, 16]. VFC protocols reorganize follow-up treatment planning by transferring part of the diagnostic phase in the emergency department (ED) to a daily supervised organized setting: a multidisciplinary VFC review meeting. Here, direct supervision is provided by an experienced orthopedic trauma surgeon and comprehensive treatment plans are discussed and scheduled from start to finish. These plans include specifics for surgical treatment (e.g. type of operation, materials needed, duration of operation, preferred timeframe), enabling administrative assistants to plan operative treatment directly within one workday after the initial ED visit.

VFC protocols have shown to reduce health care resource utilization in a variety of extremity related injuries without compromising the quality of care [15–24]. A study evaluating the implementation of a VFC review protocol for DRFs requiring non-operative treatment concluded that this approach reduced secondary healthcare utilization without increasing the risk of complications [24]. Another study evaluating the implementation of a VFC review protocol in patients with hand fractures showed a reduced time between injury and operative treatment [16]. However, the impact of the VFC on the timely scheduling of operative treatment remains largely unknown.

Implementation of a VFC review protocol could potentially be of great interest for traumatic injuries requiring operative treatment within a limited timeframe, such as DRFs. Therefore, the aim of this study was to evaluate the impact of a VFC review protocol on time between injury and surgery for patients with DRF requiring semi-acute surgical treatment, including presentation delay. Furthermore secondary healthcare utilization was assessed.

## Materials and methods

### Design and setting

This before-and-after retrospective cohort study was conducted at an urban level-2 trauma center and teaching hospital in the Netherlands. At this institution, a VFC review protocol was introduced in April 2020. All Dutch or English speaking ED patients who required follow-up treatment were reviewed according to the VFC review protocol. Exclusion criteria for VFC review were: invalid phone number, Glasgow Coma Scale < 15 at presentation, cognitive impairment, and initial treatment or follow-up elsewhere. Patients who were excluded from the VFC workflow were treated following the pre-VFC workflow protocol.

Ethical approval was obtained by the institutional review board of the hospital (WO.23.073.)

### Study population

**Fig. 1:**
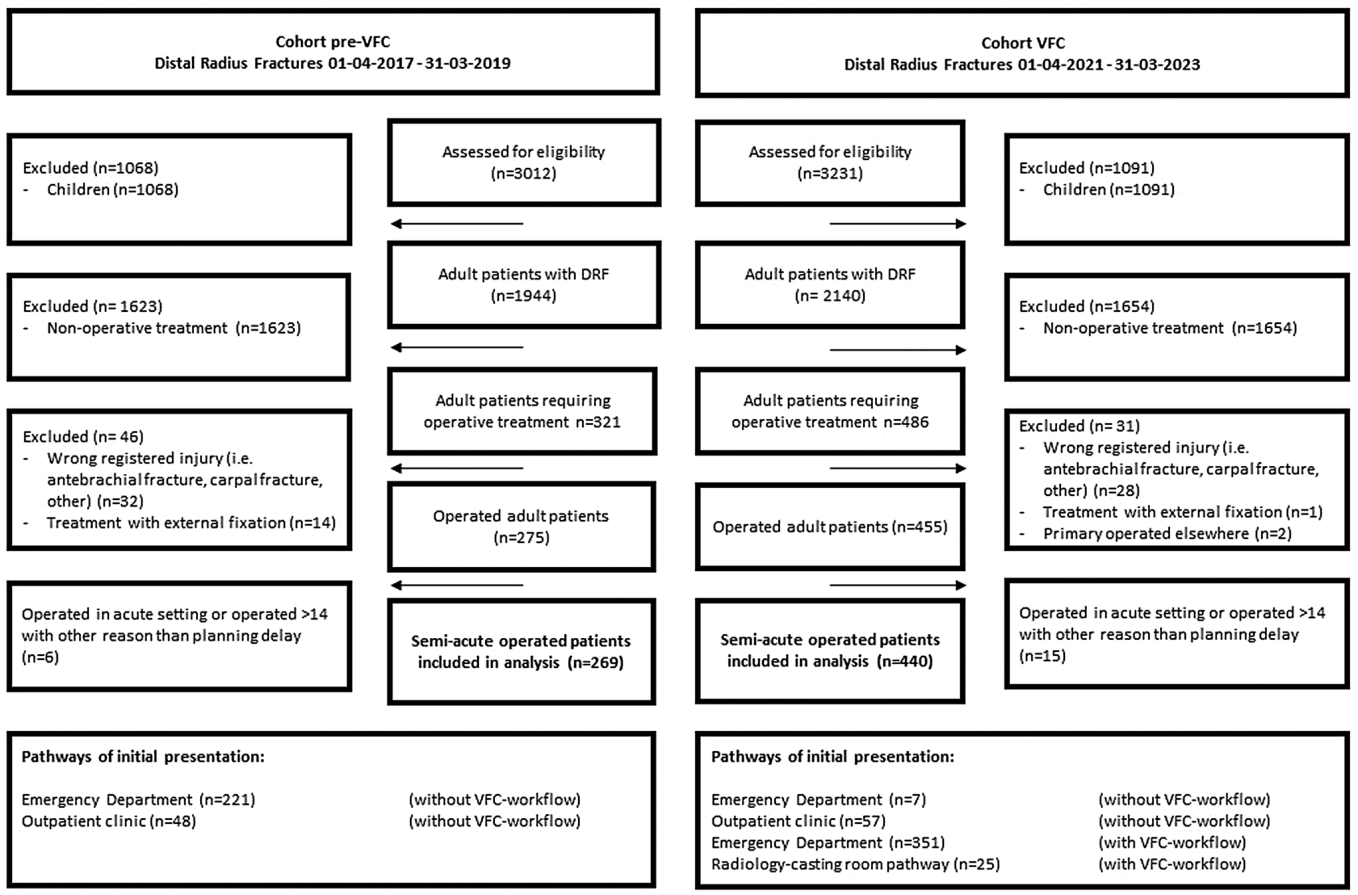
Flow diagram

Patients were included if they were diagnosed with a DRF between April 1 2017 - March 31 2019 (pre-VFC cohort) and April 1 2021– March 31 2023 (VFC cohort), if they received operative treatment with ORIF, and if operative treatment was either directly decided upon or at the first outpatient clinic follow-up visit within one week (e.g., in case of secondary displacement). Patients were excluded if they were operated in an acute setting (i.e. open fractures, nerve or vessel damage), if they were operated over 14 days due to patient delay (i.e. insurance problems, no show), if they received another type of fixation than a dorsal or volar, or dorsal and volar plate (i.e. external fixator, k-wires) (Fig. 1).

### Pre-VFC workflow

Prior to the implementation of the VFC review, treatment decisions in the ED were predominantly made by residents, with varying degrees of oversight from surgical staff. While radiographs were reviewed by the radiology department, this typically did not occur immediately after imaging. As a result, residents were initially responsible for interpreting the radiographs themselves. Treatment options were either discussed directly with the patient in the ED or deferred to an appointment at the outpatient clinic or casting room shortly after the ED visit. These follow-up appointments were usually handled by residents and a subsequent new follow-up appointment (with additional radiographs) was decided on per visit. To minimize the risk of misdiagnoses (i.e. missed (additional) fractures, underestimations of fracture severity), musculoskeletal ED radiographs from the previous day were reviewed again during a daily radiograph assessment meeting, which included a radiologist, ED staff, and an orthopaedic surgeon. If a misdiagnosis was identified, the patient was scheduled for a follow-up appointment at the outpatient clinic to discuss further treatment. Once surgery was determined as the appropriate course of action, an administrative planner would schedule the procedure and inform the patient within three working days.

### VFC review workflow

Patients with a trauma-related extremity fracture, who entered the VFC-workflow, received initial treatment at the ED or plaster room without receiving a confirmed diagnosis or a treatment strategy. On the next workday, they were discussed in a multidisciplinary review (attended by an orthopedic surgeon, surgical resident, casting technician, outpatient clinic secretary, operation planning secretary). During this VFC-review, a diagnosis was set followed by a tailor made treatment plan under direct supervision of an experienced orthopedic surgeon. For surgical treatment protocols, the VFC review pathways included information on the surgical procedure, the complete follow-up treatment and the recovery process. The VFC team could adjust treatment protocols for each patient based on expert opinion. Directly after VFC review, patients were informed by phone and consent for definitive treatment was discussed. If there was any hesitancy regarding consent or the choice of treatment, patients were scheduled for an outpatient clinic appointment to discuss treatment options. For patients needing surgery, an administrative planner scheduled the surgical procedure and all follow-up appointments once consent was obtained, and informed the patient the same day through mail or their electronic patient record. Additionally, during the VFC period, patients could also directly visit the plaster room after receiving radiological confirmation of a fracture after referral from a general practitioner. They entered the VFC-workflow.

### Data collection

Study data included age, gender, injured side, weekday of hospital presentation, number and types of hospital contacts (i.e. ED-visit, outpatient clinic visit, telephone consultation), number and type of diagnostic imaging (radiograph, MRI, CTs, ultrasound), number of immobilization materials (casts, braces or ‘other’), initial treatment (operative or non-operative), pathway of initial presentation (ED, outpatient clinic, plaster room), complexity of injury scored by a trauma surgeon pre-operatively (simple or complex) and registered complications. The time to operation was determined by calculating the difference between the date and time of the initial hospital registration and the date and time of the operation. If the injury occurred before the registration date (presentation delay), the difference in dates was added based on the history obtained during the first visit. Additionally, all patients were allotted an extra 12 hours (equivalent to 0.5 days) to account for any potential underreporting of delay between the injury and presentation.

### Data analysis

The primary outcome of this study was the exact number of days between the onset of injury and operation. A subgroup analysis was conducted excluding patients who presented through the outpatient clinic and plaster room. The plaster room route was not available in the pre-VFC period, and outpatient clinic patients were not subjected to the VFC workflow.

Additionally, the primary outcome was assessed distinctively for simple versus complex injuries, for female versus male patients, for patients in different age categories; 18–35, 36–65, 66 years and older, and descriptively for weekday of hospital presentation.

The secondary outcome was the quality of planning of semi-acute surgery; within 1–7 days after injury, within 8–14 days after injury, within 15–21 days after injury, and over 21 days after injury.

Exploratory outcomes included the number and types of hospital contacts, diagnostic imaging tests and used immobilization materials. Recorded complications were also reported.

### Statistical analysis

Baseline characteristics between the pre-VFC and VFC cohorts were compared using independent samples T-tests or Mann-Whitney U-tests for continuous data, based on data distributions. Normality of the data was assessed through visual inspection and the Shapiro-Wilk test. For normally distributed data, the mean and standard deviation were reported, while for non-normally distributed data, the median and interquartile range were provided. Categorical data were analyzed using Chi-square tests, or Fisher’s exact tests when there were zero cell counts.

The difference in days until semi-acute surgery between the pre-VFC cohort and the VFC cohort, considered the primary outcome, was assessed using the log-rank test following Kaplan-Meier survival analysis. The results were plotted as the cumulative probability of having undergone the operation. Since all patients eventually received the operation, the mean days until elective surgery along with its two-sided 95% confidence interval (95% CI) were reported. Consistency of the difference in days between injury and semi-acute surgery across weekdays of presentation was described in the Supplemental material.

Cox regression analyses were conducted to further quantify the impact of VFC implementation by estimating the corresponding mean hazard ratio (HR) for elective surgery following DRF along with its bias-corrected and accelerated 95% non-parametric confidence interval (BCa 95% CI) following bootstrapping, drawing 1000 samples of the same sizes as the original samples with replacement, stratified by VFC-period [25, 26]. Similarly, Cox regression analyses were performed to estimate the HR for semi-acute surgery after substratification by injury severity, gender, and age category respectively. Difference in the secondary outcome quality of timely surgical planning was detected using Chi-square tests or Fisher’s exact tests. A p-value < 0.05 indicated statistical significance for differences in primary and secondary outcomes for DRFs. Exploratory outcomes were documented descriptively.

## Results

### Patients and baseline characteristics

In total, 269 were included in the pre-VFC cohort and 440 in the VFC cohort (Fig. 1). Of all patients, the median age was 57 years (IQR 39–66), 58% injured the left hand, and 70% were female. Female (median 60, IQR 48–68) were older than males (median 45, IQR 32–57) (*p* < 0.001).

Before VFC implementation, 29% of all semi-acute operated patients commenced with a non-operative treatment plan and shifted to operative treatment at their first follow-up. Following VFC implementation, this figure decreased to 11% (*p* < 0.001). Of the 440 patients included in the VFC cohort, 376 were treated with the VFC workflow (compliance of 86%) (Table 1).

**Table 1:** Baseline characteristics

	Semi-acute operated distal radius fractures		*p*-value
	Pre-VFC (*n* = 269)	VFC (*n* = 440)	
Age in years: Median (IQR)	57 (39–66)	57 (38–66)	0.87
Female: n (%)	198 (74)	300 (68)	0.13
Left-sided injuries: n (%)	152 (57)	256 (58)	0.66
Simple injuries: n (%)	171 (64)	254 (58)	0.12
Initially treated operatively: n (%)	190 (71)	391 (89)	< 0.001
Presentation delay in days Median (IQR)	0 (0–1)	0 (0–1)	0.112
Route			
Emergency department (%)	221 (82)	358 (81)	
Out-patient clinic (%)	48 (18)	57 (13)	
Radiology/Plaster room (%)	Not applicable	25 (6)	

ED: emergency department, VFC: virtual fracture clinic

### Primary outcome

**Fig. 2:**
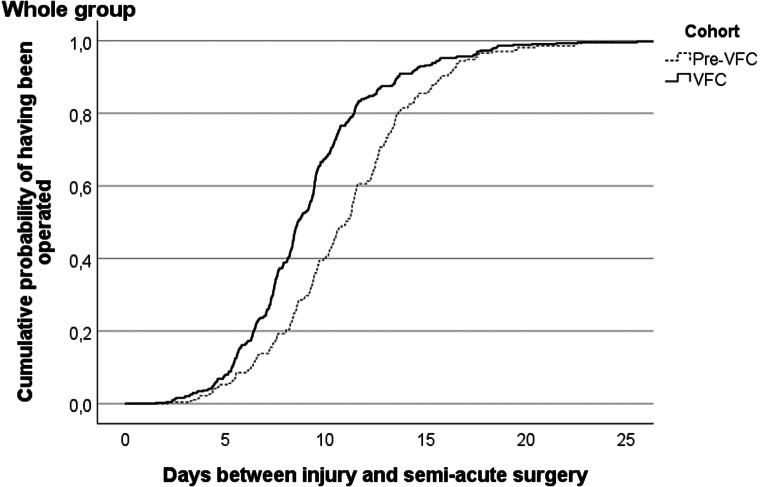
Cumulative probability for patients by VFC-cohort of having received semi-acute surgery following day of injury with a distal radius fracture

Figure 2 illustrates the cumulative probability of undergoing semi-acute surgery based on the number of days since injury. In the pre-VFC cohort, an average of 11.0 days elapsed before surgery for 269 patients. Following VFC implementation, the average time to surgery significantly decreased to 9.2 days for 440 patients (*p* < 0.001). See also Supplemental Material Table S1 for distinct weekdays of presentation.

The HR for semi-acute surgery in the VFC cohort, considering the entire group, was 1.6 (BCa 95% CI: 1.3–1.8), indicating an increased probability by 60% in the VFC cohort of receiving semi-acute surgery earlier compared to the pre-VFC cohort (Table 2).

**Table 2:** Time between injury and semi-acute surgery by treatment group and corresponding hazard ratio’s

	Pre-VFC	VFC	Log Rank	*p*-value	Hazard Ratio
	Days **(**95% CI; N)	Days **(**95% CI; N)			mean (BCa 95% CI)
All adults	11.0 (10.6–11.5; 269)	9.2 (8.9–9.6; 440)	32.8	< 0.001	1.6 (1.3–1.8)
*ED-visit only*	10.6 (10.1–11.1; 221)	8.5 (8.2–8.7; 358)	50.9	< 0.001	1.8 (1.5–2.3)
Simple injury	10.8 (10.2–11.3; 171)	9.1 (8.7–9.5; 254)	18.2	< 0.001	1.5 (1.2-2.0)
Complex injury	11.5 (10.7–12.3; 98)	9.3 (8.8–9.9; 186)	14.2	< 0.001	1.6 (1.3–2.1)
Female	11.3 (10.7–11.8; 198)	9.2 (8.8–9.6; 300)	30.9	< 0.001	1.7 (1.4-2.0)
Male	10.4 (9.6–11.2; 71)	9.3 (8.6–9.9; 140)	4.2	0.041	1.3 (1.0-1.8)
Aged 18–35	10.0 (9.1–10.9; 59)	9.6 (8.8–10.5; 100)	0.7	0.388	1.2 (0.8–1.6)
Aged 36–65	10.9 (10.3–11.6; 137)	8.9 (8.4–9.3; 224)	22.5	< 0.001	1.7 (1.3–2.1)
Aged 66+	12.0 (11.1–13.0; 73)	9.5 (8.9–10.1; 116)	16.8	< 0.001	1.9 (1.4–2.6)

ED: emergency department

After excluding patients who presented through the outpatient clinic or plaster room, the remaining patients (*N* = 579) were operated on after an average of 10.6 days before and 8.5 days in the VFC-period, with a HR of 1.8 (BCa 95% CI: 1.5–2.3) (Table 2).

Table 2 further signifies that decreases were observed across subgroups of patients by type of injury, gender and age, except for the younger adults between 18 and 35 years of age. Probabilities of having been operated earlier ranged from 35 to 86%. See also Supplemental material Figures S1-S3.

### Secondary outcome

Before VFC implementation, semi-acute surgery was performed within 7 days after injury in 17% of patients, and within 14 days in 84%. After the implementation of VFC, 33% of patients received surgery within 7 days, and 92% within 14 days. Surgery performed in the week three was reduced from 14 to 7% with the implementation of VFC. Before VFC implementation 1.5% of patients received semi-acute surgery beyond the third week against 0.9% in the VFC-period. VFC-implementation improved the quality of timely planning (Pearson Chi2 = 27.4; *p* < 0.001).

### Exploratory outcomes

The median number of total hospital contacts per patient before VFC implementation was 5 (IQR: 4–6) versu 4 after VFC implementation(IQR: 3–5). The total number of physical visits at the outpatient clinic decreased from 4 (IQR: 3–5) to 1 (IQR: 1–2) while the number of telephone consults was negligible before VFC implementation and increased to a median of 1 (IQR: 1–2) after implementation. While the median number of radiographs per patient dropped from 6 (IQR: 5–7) to 4 (IQR: 3–5), the number or CT-scans executed was comparable: 1 (IQR: 0–1) versus 1 (IQR: 1–1), frequencies of imaging with MRI 0 (IQR: 0–0) and ultrasound 0 (IQR: 0–0), stayed equally low after VFC implementation. The median number of casts remained stable at 1 (IQR: 1–2).

## Discussion

This study assessed the impact of implementation of a VFC review protocol on time between injury and semi-acute surgery for patients with DRFs. Furthermore the study aimed to assess potential reductions in secondary health care utilization.

The introduction of a VFC review protocol was associated with a mean decrease of 1.8 days between injury and operation and an increase of 60% in the probability of being operated earlier during the first 3 weeks after injury with a DRF. When considering solitary patients presenting through the ED, an even greater reduction of 2.1 days in the time between injury and semi-acute surgery was observed.

This improvement was robust across simple and complex injury types, 1.7 and 2.2 days respectively. Before the implementation of the VFC, simple injuries were operated on about a day earlier than complex injuries. After VFC implementation, the average time to operation for both simple and complex injuries became equal. Complex injuries typically require more operation time and often necessitate the expertise of a more experienced surgeon, making them more difficult to schedule. A plausible reason for the alignment in surgery times after VFC implementation could be found in an improved quality of planning.

Female patients, particularly elderly women, constitute the majority of the population with DRF, a trend consistent with findings in the literature [2, 4, 6–8]. Female patients benefited more from the implementation of the VFC, with a reduction of 2.1 days compared to a reduction of 1.1 days for male patients, because before the implementation, male patients were, on average, operated on within 10.4 days, whereas female patients were operated on within 11.3 days.

Patients aged 18–35 did not experience significant benefits from VFC implementation, as their time to operation was already shorter compared to all elderly patients. Within this age category, males comprised 47% of all patients. Patients aged 36–65 showed a significant improvement, with a reduction of 2.0 days, and males represented 33% of this group. Last, patients aged 66 and older benefited the most, with a reduction of 2.5 days and a 90% increase in the probability of being operated on within three weeks. Only 11% of this age group was male.

Regardless of the underlying causes for differences in the pre-VFC setting, the implementation of VFC has demonstrated its effectiveness in equalizing disparities between (1) simple and complex injuries, (2) female and male patients, and (3) different age categories and in significantly reducing the overall time until surgery.

After the implementation of VFC, one-third of patients underwent surgery within the first week, more than 90% within the second week, and in 99 out of 100 patients within three weeks. Notably, the median presentation delay between injury and hospital presentation was 5 days and 19 days in respectively patients operated within 2–3 weeks and over 3 weeks. Given the absence of high-quality, evidence-based studies assessing patient outcomes related to time-to-surgery, our study findings are favorable. Literature does not offer clear-cut guidance on operating within 2–3 weeks. Nevertheless, the majority of publications and international guidelines support that a distal radius fracture operated on within 2 weeks is certainly acceptable, indicating a compliance rate of 92% with our study outcomes [1, 6, 8–13].

The exploratory outcomes support the assumption that the implementation of VFC reduced the pressure on the health care system and thereby reduces direct health care costs [15, 17–24]. The total median number of hospital contacts decreased from 5 to 4, although more interestingly, the physical contacts with a physician decreased from 4 to 1 in exchange for one 5 min telephone consultation. Furthermore, the median number of radiographs was reduced from 6 to 4. These positive effects are appointed to supervision of an authorized specialist on the VFC review, where an adequate diagnosis can be directly established, followed by a beginning-to-end treatment plan, which reduces unnecessary follow-ups, radiographs or treatment conversions (29.4% versus 11.1% initially started non-operatively (*p* = < 0.001)) [24]. The median number of casts per patient was 1, supporting the Dutch guideline that after operative fracture fixation, a cast is no longer needed and movement and mobilization of the wrist is feasible [10].

A key strength of this study lies in its evaluation of “time between injury and semi-acute surgery” in its actual state, all participating pathways included, which provides a pragmatic assessment of outcomes. With an 86% compliance of the VFC workflow in the VFC implementation cohort, this study acknowledges the current limitation of achieving full compliance with the VFC workflow at this stage. The findings of this study can be applied to real-world clinical scenarios.

Another strength is the allowance for an overestimation in time between injury and surgery of up to 12 hours (equivalent to 0.5 days) to account for any potential delay between the injury and actual presentation. This likely results in a shorter actual “time to surgery.”

The last strength of the study is its substantial sample size.

A limitation of this study is its retrospective design, which carries an inherent risk of bias. Additionally, the absence of an external control group in this before-and-after study prevents us from distinguishing between background trends or external developments over time.

## Electronic supplementary material

Below is the link to the electronic supplementary material.

Supplementary Material 1

Supplementary Material 2

Supplementary Material 3

Supplementary Material 4

Supplementary Material 5

Supplementary Material 6

Supplementary Material 7

Supplementary Material 8

Supplementary Material 9

## Data Availability

Data are available upon reasonable request to the corresponding author.
