# Supplementary figures and images for: Efficiency of a virtual fracture clinic review protocol in adult patients with distal radial fractures requiring semi-acute surgical treatment
Source: Eur J Trauma Emerg Surg. 2025 Feb 7;51(1):96. doi: 10.1007/s00068-025-02764-3 (PMC11805890; doi:10.1007/s00068-025-02764-3)

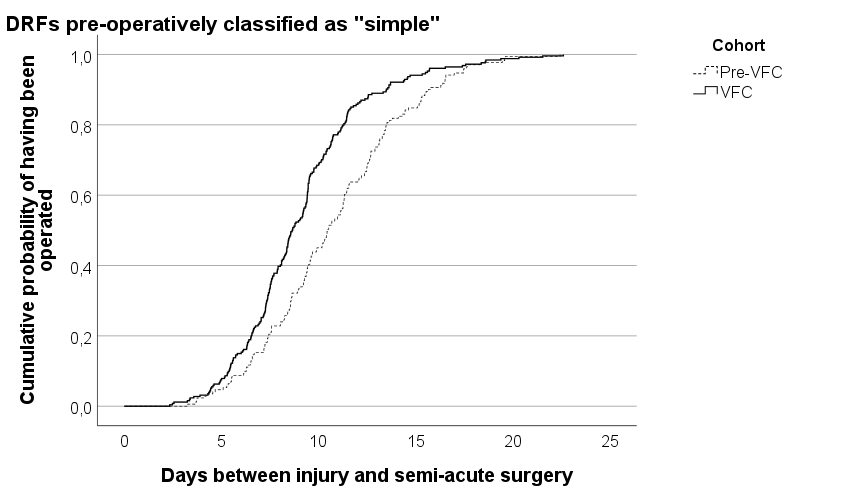

Supplement: Supplementary file 1 — Supplementary Material 1 [file 68_2025_2764_MOESM1_ESM.png]

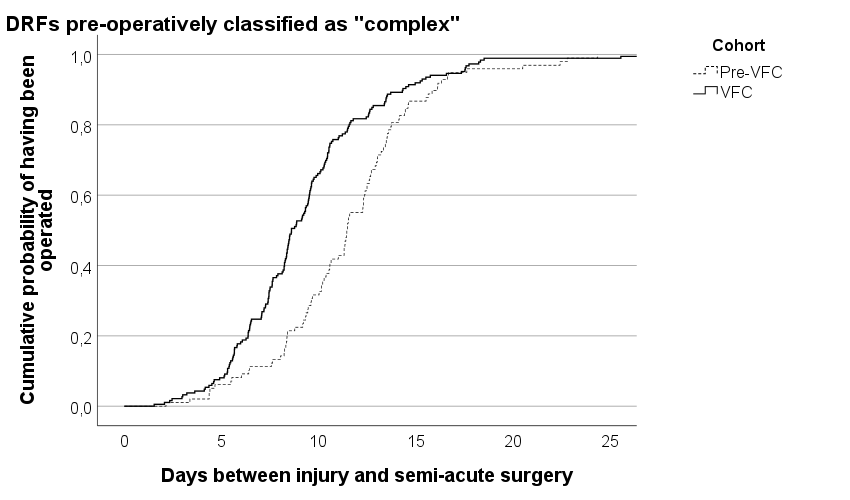

Supplement: Supplementary file 2 — Supplementary Material 2 [file 68_2025_2764_MOESM2_ESM.png]

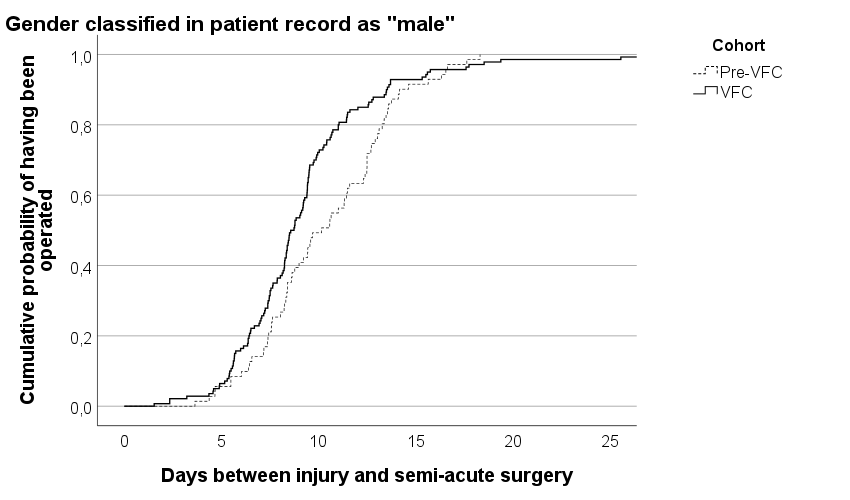

Supplement: Supplementary file 3 — Supplementary Material 3 [file 68_2025_2764_MOESM3_ESM.png]

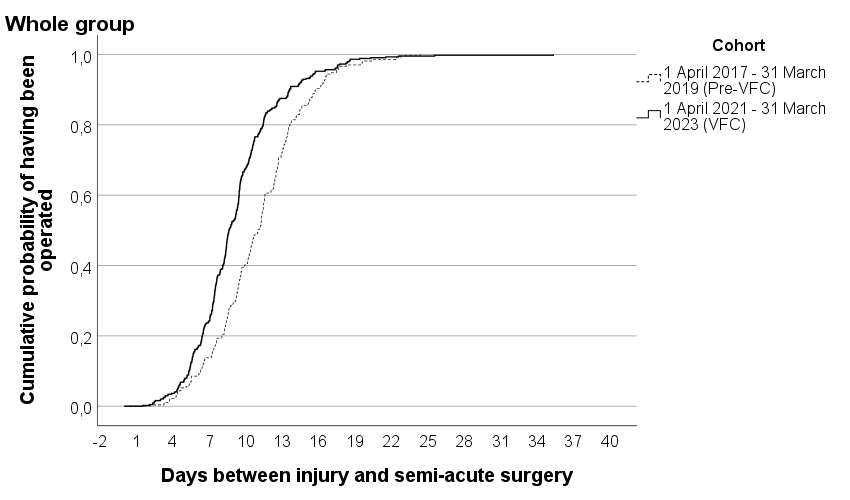

Supplement: Supplementary file 4 — Supplementary Material 4 [file 68_2025_2764_MOESM4_ESM.png]

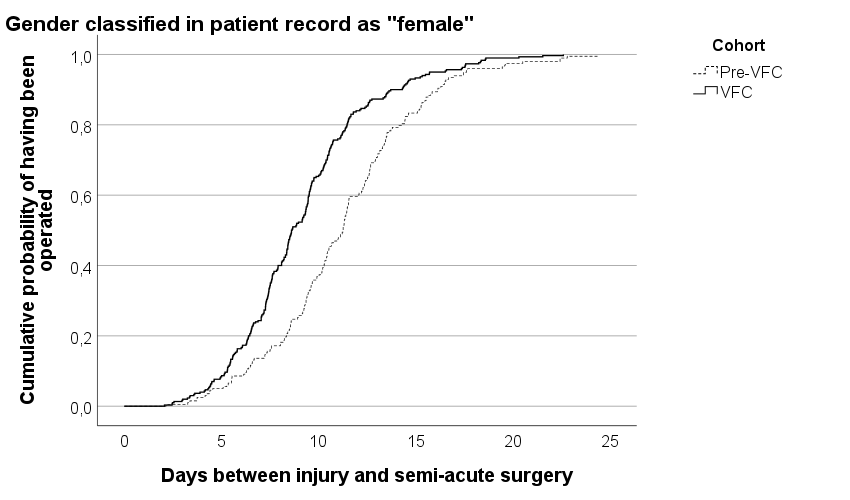

Supplement: Supplementary file 5 — Supplementary Material 5 [file 68_2025_2764_MOESM5_ESM.png]

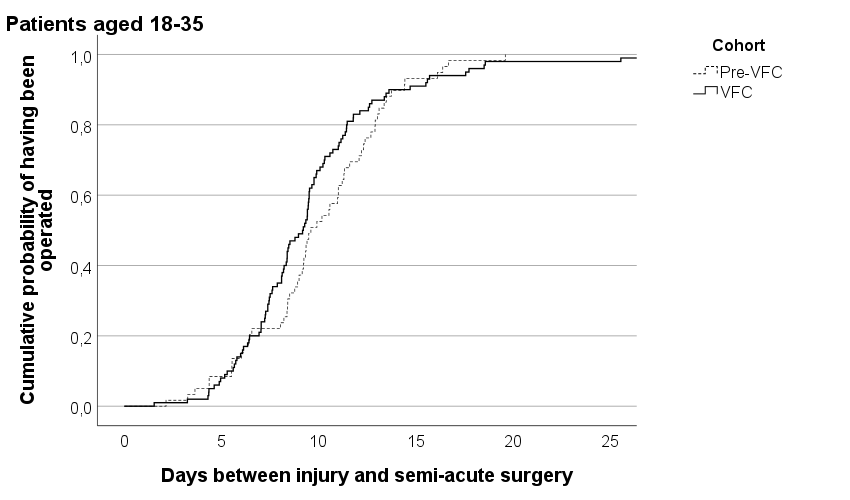

Supplement: Supplementary file 6 — Supplementary Material 6 [file 68_2025_2764_MOESM6_ESM.png]

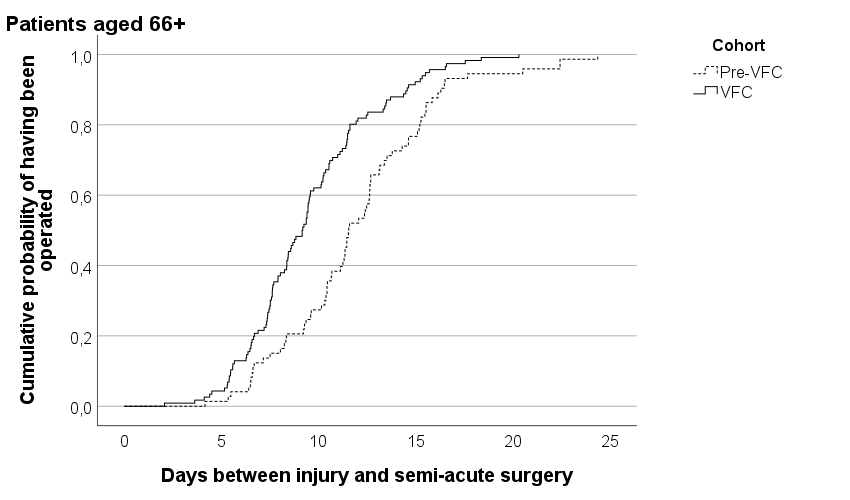

Supplement: Supplementary file 7 — Supplementary Material 7 [file 68_2025_2764_MOESM7_ESM.png]
