# Supplementary material for: Efficiency of a virtual fracture clinic review protocol in adult patients with distal radial fractures requiring semi-acute surgical treatment
Source: Eur J Trauma Emerg Surg. 2025 Feb 7;51(1):96. doi: 10.1007/s00068-025-02764-3 (PMC11805890; doi:10.1007/s00068-025-02764-3)
Supplement: Supplementary file 8 — Supplementary Material 8 [file 68_2025_2764_MOESM8_ESM.docx]

Table S1. Time between injury and semi-acute surgery by treatment group by weekday of presentation

| **Weekday of presentation** | **Pre-VFC** | **VFC** |
| --- | --- | --- |
| *General* | *11.0 (10.6-11.5; N=269)* | *9.2 (8.9-9.6; N=440)* |
| Monday | 11.1 (10.1-12.2; N=56) | 10.0 (8.9-11.1; N=82) |
| Tuesday | 10.8 (9.5-12.1; N=38) | 9.5 (8.6-10.4; N=63) |
| Wednesday | 11.1 (9.6-12.5; N=25) | 9.0 (7.9-10.0; N=48) |
| Thursday | 11.1 (10.1-12.1; N=48) | 9.0 (8.1-10.0; N=52) |
| Friday | 11.3 (9.9-12.6; N=35) | 9.8 (9.1-10.5; N=85) |
| Saturday | 10.9 (9.7-12.1; N=41) | 8.3 (7.6-9.0; N=50) |
| Sunday | 11.0 (9.8-12.2; N=26) | 8.1 (7.4-8.8; N=60) |
