# Supplementary material for: Efficiency of a virtual fracture clinic review protocol in adult patients with distal radial fractures requiring semi-acute surgical treatment
Source: Eur J Trauma Emerg Surg. 2025 Feb 7;51(1):96. doi: 10.1007/s00068-025-02764-3 (PMC11805890; doi:10.1007/s00068-025-02764-3)
Supplement: Supplementary file 9 — Supplementary Material 9 [file 68_2025_2764_MOESM9_ESM.docx]

**SUPPLEMENTARY MATERIAL**

*Figure S1. Cumulative probability for patients by VFC-cohort of having received semi-acute surgery following day of injury with a distal radius fracture pre-operatively classified as a) simple and b) complex*

*Figure S2. Cumulative probability by VFC- cohort of having received semi-acute surgery following day of injury with a distal radius fracture in a) female patients and b) male patients*

*Figure S3. Cumulative probability by VFC-cohort of having received semi-acute surgery following day of injury with a distal radius fracture in patients aged a) 18-35, b) 36-65, and c) 66 years and older.*

*Table S1. Time between injury and semi-acute surgery by treatment group by weekday of presentation*
